# Prevalence of perinatal depression among Japanese men: a meta-analysis

**DOI:** 10.1186/s12991-020-00316-0

**Published:** 2020-11-18

**Authors:** Keita Tokumitsu, Norio Sugawara, Kazushi Maruo, Toshihito Suzuki, Norio Yasui-Furukori, Kazutaka Shimoda

**Affiliations:** 1 Department of Psychiatry, Dokkyo Medical University School of Medicine, Mibu, Shimotsuga, Tochigi 321-0293 Japan; 2 Department of Neuropsychiatry, Towada City Hospital, Towada, Japan; 3 Department of Clinical Epidemiology, Translational Medical Center, National Center of Neurology and Psychiatry, Kodaira, Japan; 4 Department of Biostatistics, Faculty of Medicine, University of Tsukuba, Ibaraki, Japan; 5 Department of Psychiatry, Juntendo Koshigaya Hospital, Saitama, Japan; 6 Department of Neuropsychiatry, Graduate School of Medicine, Hirosaki University, Hirosaki, Japan

**Keywords:** Perinatal depression, Prenatal depression, Postpartum depression, Father, Men, Japanese

## Abstract

**Introduction:**

Perinatal depression is a widely discussed mental illness that occurs not only in women, but also in men. A previous international meta-analysis of the prevalence of paternal perinatal depression suggested that cross-cultural variables or socioeconomic environment may influence paternal depression. However, it is not clear that these data are general enough to apply to Japanese men, and there are few review articles about perinatal depression among this demographic. The purpose of our study is to provide a reliable estimate of the prevalence of perinatal depression among Japanese men.

**Method:**

We searched two databases, PubMed and ICHUSHI, to identify studies with data on the prevalence of prenatal or postpartum depression among Japanese men. Data were extracted from reports published from January 1994 to June 2018. The period prevalence of paternal perinatal depression among Japanese men was investigated. A subgroup analysis of gender differences in perinatal depression was also performed.

**Results:**

We reviewed 1,379 abstracts, retrieved 33 articles and ultimately included 15 studies. The period prevalence of paternal prenatal depression in men was 8.5% (95% CI 3.3–20.3%). Moreover, the period prevalence of postpartum depression in men was 9.7% (95% CI 7.4–12.8%) within the first month, 8.6% (95% CI 5.5–13.3%) in postpartum months 1–3, 13.2% (95% CI 11.6–15.0%) in postpartum months 3–6 and 8.2% (95% CI 1.3–38.0%) in postpartum months 6–12. We also found that the prevalence of prenatal depression was significantly lower in men than in women. However, the prevalence of postpartum depression was not significantly different between men and women.

**Conclusions:**

The prevalence of perinatal depression among Japanese men peaked at 3–6 months after birth, and its overall prevalence was approximately 10%. These results were similar to those of an international meta-analysis on perinatal depression. Notably, we found that the prevalence of postpartum depression was as high in men as it was in women. Therefore, it is suggested that healthcare workers should be more watchful for paternal perinatal depression in the postpartum period than in the prenatal period.

## Introduction

Perinatal depression, a mental illness that occurs either during pregnancy or within the first 12 months after delivery, affects the health and development of mothers and children [1, 2]. Accordingly, societal views emphasize that men must help perinatal women and children [3]. On the other hand, paternal perinatal depression is a mood disorder among men that occurs in the perinatal period of their female partners [4, 5]. Recently, men have also been found to develop perinatal depression at high rates [4]. Notably, suicide risk increases with perinatal depression among not only women, but also men [6]. It has been reported that maternal and paternal perinatal depression are correlated [7]. Accordingly, it is necessary for women and men to evaluate their mental states and seek appropriate assistance during the perinatal period. In Japan, it was found that maternal mortality was influenced more strongly by suicide related to maternal postpartum depression than by perinatal physical complications [8]. In order to prevent suicide in the parents and promote the healthy development of the child, family care is important. Additional studies have shown that maternal postpartum depression carries a risk of adverse effects on the child, such as emotional disturbances and developmental disorders [1, 9]. Unfortunately, 133,778 cases of child abuse were reported from April 2017 to March 2018 [10]. In addition, 77 children died because of child abuse from April 2016 to March 2017 [10]. In Japan, the number of children who suffer from abuse is increasing yearly, creating a major social problem [10]. Several studies have shown that maternal perinatal depression is a risk factor for child abuse [11, 12]. In recent years, it has been found that paternal postpartum depression is associated with an increased risk of child maltreatment in Japan [13]. Thus, it has been suggested that early detection and early treatment of maternal and paternal postpartum depression are useful for preventing child abuse. Therefore, perinatal mental illness is an important issue in Japan from the viewpoint of public health.

In recent years, screening tests for depression have been conducted for Japanese mothers in the perinatal period [14]. Japanese women suffering from postpartum depression often have mental problems during pregnancy [14]. Thus, early intervention in pregnant women is advisable to prevent suicide due to perinatal depression. According to a meta-analysis of studies that used evaluation scales to assess patients experiencing perinatal depression, the prevalence of female perinatal depression was reported to be approximately 13–19% [15]. However, it is difficult to identify all the causes and consequences because the disease is highly heterogeneous and involves biological, psychological and social factors intertwined in complex ways [15]. Recently, the international prevalence of perinatal depression in men was also meta-analyzed, and the prevalence was reported to be 8–13% [4, 16]. Furthermore, perinatal depression in men is also highly heterogeneous, and a previous report also suggested that cross-cultural variables or the socioeconomic environment may influence paternal depression [4].

In order to more accurately estimate the prevalence of perinatal depression, it is necessary to consider the heterogeneity of the disease etiology. When considering men’s mental health problems, we should consider cultural and social background. The OECD reports that Japanese men spend more time on paid labor than men in Western countries do. For this reason, the amount of time that men spend with their children and wives at home may be shorter in Japan than in other countries [17]. According to a survey by the Ministry of Internal Affairs and Communications, men (husbands) with a child under 6 years old spent 49 min per day on childcare [18]. This is only 20% of the amount of time spent by women (wives) [18]. As a result, especially in the perinatal period, wives may become depressed due to isolation in the home.

Additionally, there is a cultural practice called "satogaeri" childbirth in Japan [19]. This practice instills the expectation that a pregnant woman will leave her husband and spend the perinatal period with her parents, thereby reducing her psychological anxiety and physical burden [20]. However, this practice reduces the time available for the formation of a father–child bond and decreases the number of conversations between the partners [21]. Hence, there is a risk that men will be isolated. Thus, Japanese men have different cultural characteristics than men in other countries. Based on this social environment, we performed a meta-analysis of the prevalence of perinatal depression specifically in Japanese men. Additionally, there is little literature reviewing the differences between men and women regarding the prevalence of perinatal depression. Therefore, we extracted data from previous publications to calculate the prevalence of perinatal depression among couples within the same study and examined the relative risk of depression before and after childbirth.

## Methods

### Study selection

This systematic review is reported according to the Preferred Reporting Items for Systematic Reviews and Meta-Analyses (PRISMA) standards (a protocol used to evaluate systematic reviews) [22]. We searched for published studies related to perinatal depression in two electronic databases: PubMed and ICHISHI. The search query for PubMed was ((pregnancy [ALL] OR antenatal [ALL] OR prenatal [ALL] OR gestation [ALL] OR postnatal [ALL] OR postpartum [ALL] OR postpartal [ALL] OR perinatal [ALL] OR puerperium [ALL] OR puerperal [ALL] OR postbirth [ALL] OR post-birth [ALL]) AND (depression [ALL] OR depressive [ALL] OR mood disorder [ALL] OR affective disorder[ALL]) AND (Japan [ALL] OR Japanese [ALL]) AND (1994/1/1: 2018/6/30 [DP])).

In addition, the ICHUSHI (http://search.jamas.or.jp/) database was searched for articles written in Japanese. ICHUSHI contains bibliographic citations and abstracts from biomedical journals and other serial publications published in Japan. We searched ICHUSHI using Japanese search terms comparable to the English PubMed search terms, but without the terms “Japan” and “Japanese”.

Two electronic databases, PubMed and ICHUSHI, were searched for studies published from January 1, 1994, to June 30, 2018. The Diagnostic and Statistical Manual of Mental Disorders—Fourth Edition (DSM-IV) [23] was the first diagnostic manual to specify postpartum onset of major depressive disorder. Hence, we excluded older studies that were published before the release of the DSM-IV. Then, we examined the lists of references included in the articles.

### Inclusion and exclusion criteria

Studies were eligible for inclusion if they (a) included men; (b) assessed antenatal or postpartum depression using a validated self-report instrument; and (c) reported data to estimate the prevalence of antenatal or postpartum depression. Studies were excluded if they (a) recruited only high-risk populations (e.g., patients with a history of mental illness); (b) reported results for only a subsample of a study population; (c) reported duplicate data from a single database; (d) reported only mean data; or (e) did not report a cutoff point for depression scales. For studies with duplicate data from a single database, we selected the study with the larger sample size.

Case reports, comments, editorials, letters, and studies not performed on human participants were also excluded. Two researchers (KT and NS) independently screened the literature. After all papers had been assessed, any discrepancies in the responses were identified and discussed to reach a consensus on the best option. Disagreements about the inclusion of a study were resolved through discussion with the senior author (NYF). Data were extracted from each article using a standardized form that recorded the first author, publication year and other information.

### Data extraction

From each study, we extracted information about the publication year, the sample size, the measures used to assess depression, the cutoff point used for each measure, the time points of depression assessment, and the prevalence rates of prenatal and postpartum depression.

The time of measurement was defined as during the prenatal period, within 1 month postpartum, > 1 to 3 months postpartum, > 3 to 6 months postpartum, or > 6 months to 1 year postpartum. Moreover, only the baseline data were extracted from interventional studies. If data were extracted from longitudinal studies, only the rate from one time point in each period (e.g., prenatal or postpartum) was included in the analyses. For most studies, the first time point was used, as the participants were least familiar with the study tool at that point and were unlikely to exhibit priming effects.

The Edinburgh Postnatal Depression Scale (EPDS) and the Postpartum Depression Screening Scale completely exclude somatic symptoms and are technically validated for the pregnancy and postpartum period [24]. Therefore, when multiple evaluation scales were used within the same study, we chose the EPDS and the Postpartum Depression Screening Scale over other evaluation scales.

### Statistical analysis

First, we assessed the pooled prevalence of paternal perinatal depression during each period. We then calculated the relative risk to investigate the differences in the prevalence of perinatal depression between men and women. We used the *I*^*2*^ statistic and its 95% CI to estimate heterogeneity. The *I*^*2*^ statistic was considered high when it was 75% or higher [25]. The significance level was set at *p* < 0.05. The meta-analysis and related statistical analysis were performed with the meta package (version 4.9-1) in R (version 3.5.0). Sensitivity analysis was performed by excluding individual studies and reanalyzing the remaining data to evaluate the robustness of the data.

## Results

### Search results and included participants

**Fig. 1:**
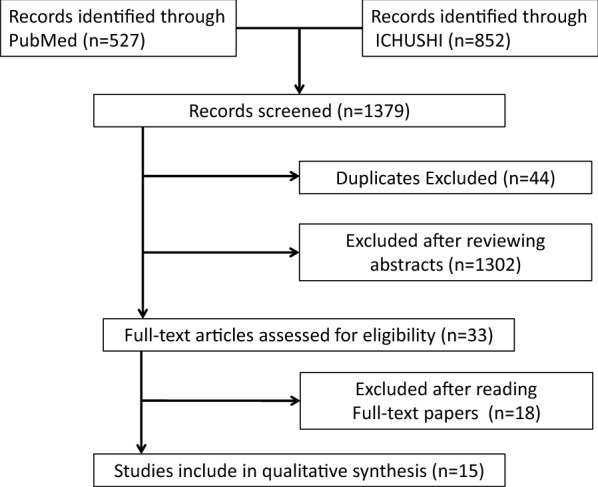
Flowchart of the process of selecting studies for inclusion

We found 15 publications that met the inclusion criteria (Fig. 1). The mean age of participants in each study was 32.0 to 37.0 years. All studies included in this meta-analysis screened the general population, not the specific population patients who visited the hospital suffering mental illness. The sample sizes of the studies ranged from 42 to 41,506 men. Among these publications, the study by Konishi used the 6-question and 10-question versions of the Kessler Screening Scale for Psychological Distress (K6 and K10, respectively) and the PHQ-9 to assess the same group of participants at the same time [26]. The PHQ-9 was developed as a tool to assess the physical and psychiatric symptoms of patients who consulted a primary care physician [27]. Therefore, it was judged that the K6 or K10 was more suitable than the PHQ-9 for screening the general population. A previous study reported that the K6 was more useful than the K10 [28]; therefore, the K6 was used for our meta-analysis of the study by Konishi. The data from the other publications could be sorted according to the eligibility criteria. Further details on the included studies and participants [13, 26, 29–41] are presented in Table 1.

**Table 1:** Major characteristics of studies on the prevalence of perinatal depression

Author, year	Design	Sex	Measure	Cutoff	Time classification for moderation analysis	Cases	Sample size	Prevalence %
Enya 2018	Longitudinal study	Men	EPDS	8/9	Within 1 month postpartum	8	218	3.7
Enya 2018	Longitudinal study	Women	EPDS	8/9	Within 1 month postpartum	38	306	12.4
Fujita2014	Cross-sectional	Men	EPDS	7/8	3–6 months postpartum	103	726	14.2
Fujita2014	Cross-sectional	Women	EPDS	8/9	3–6 months postpartum	73	726	10.1
Fukuoka 2016	Cross-sectional	Men	CES-D	15/16	Within 1 month postpartum	6	44	13.6
Fukuoka 2016	Cross-sectional	Women	CES-D	15/16	Within 1 month postpartum	7	44	15.9
Hagino 2006	Baseline data	Women	SDS	42/43	Prenatal period	17	53	32.1
Hagino 2006	Baseline data	Men	SDS	39/40	Prenatal period	8	53	15.1
Hamazaki 2017	Cohort study	Women	K6	12/13	Prenatal period	2527	75,139	3.4
Hamazaki 2017	Cohort study	Men	K6	12/13	Prenatal period	776	41,506	1.9
Higai 2008	Cross-sectional	Men	EPDS	8/9	Within 1 month postpartum	22	166	13.3
Iwafuji 2007	Longitudinal study	Men	CES-D	15/16	6–12 months postpartum	25	129	19.4
Iwafuji 2007	Longitudinal study	Women	CES-D	15/16	6–12 months postpartum	20	129	15.5
Iwafuji 2007	Longitudinal study	Men	CES-D	15/16	3–6 months postpartum	21	129	16.3
Iwafuji 2007	Longitudinal study	Women	CES-D	15/16	3–6 months postpartum	13	129	10.1
Iwafuji 2007	Longitudinal study	Men	CES-D	15/16	Prenatal period	30	217	13.8
Iwafuji 2007	Longitudinal study	Women	CES-D	15/16	Prenatal period	47	228	20.6
Konishi 2016	Cross-sectional	Men	K6	4/5	Prenatal period	14	136	10.3
Nishigori 2016	Cohort study	Men	K6	12/13	6–12 months postpartum	48	1470	3.3
Nishigori 2016	Cohort study	Men	EPDS	7/8	3–6 months postpartum	157	1370	11.5
Nishigori 2016	Cohort study	Men	EPDS	7/8	Within 1 month postpartum	122	1064	11.5
Nishimura 2010	Cross-sectional	Men	EPDS	7/8	Within 1 month postpartum	17	146	11.6
Nishimura 2010	Cross-sectional	Women	EPDS	8/9	Within 1 month postpartum	50	178	28.1
Nishimura 2015	Cross-sectional	Men	EPDS	7/8	3–6 months postpartum	110	807	13.6
Nishimura 2015	Cross-sectional	Women	EPDS	8/9	3–6 months postpartum	83	807	10.3
Suto 2016	Longitudinal study	Men	EPDS	7/8	1–3 months postpartum	17	195	8.7
Suto 2016	Longitudinal study	Men	EPDS	7/8	Within 1 month postpartum	18	207	8.7
Takagi 2017	Longitudinal study	Men	CES-D	15/16	Within 1 month postpartum	3	42	7.1
Takagi 2017	Longitudinal study	Men	CES-D	15/16	Prenatal period	5	42	11.9
Takehara 2017	Longitudinal study	Men	EPDS	7/8	1–3 months postpartum	18	209	8.6
Takehara 2017	Longitudinal study	Men	EPDS	7/8	Within 1 month postpartum	18	228	7.9
Takehara 2017	Longitudinal study	Men	EPDS	7/8	Prenatal period	26	270	9.6
Watabe 2016	Cross-sectional	Men	EPDS	8/9	Prenatal period	5	73	6.8

We found that the research conducted in Nishio City was published in two different papers (Takehara [13] and Suto [40]) and included duplicate participants. Table 1 records the data extracted from each study, but we included only reports with larger sample sizes in the meta-analysis, and Suto [40] was excluded.

Prevalence of paternal perinatal depression, test of heterogeneity and publication bias.

**Fig. 2:**
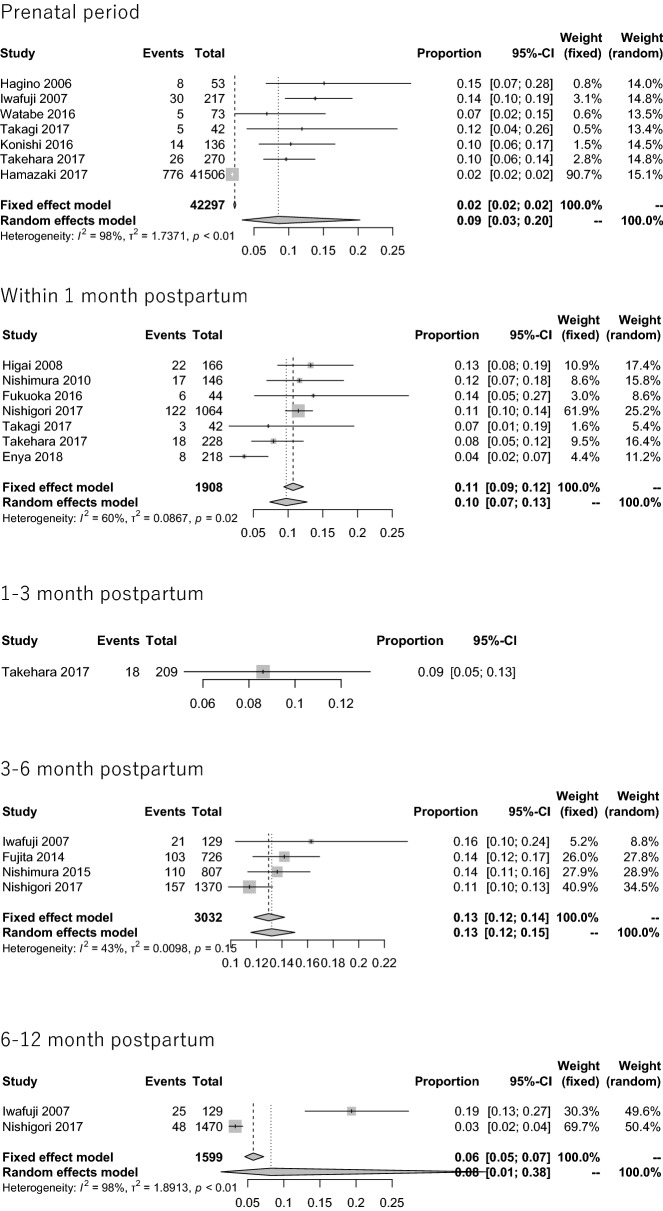
Forest plot for paternal perinatal depression among Japanese men

The period prevalence of paternal prenatal depression was 8.5% (95% CI 3.3–20.3%, *Q* = 260.61, *p* < 0.0001, *I*^2^ = 97.7%, tau^2^ = 1.737), as determined from the data included in 7 papers. A visual inspection of the funnel plot for the period encompassing the first month revealed asymmetry, and Egger’s regression test for funnel plot asymmetry was statistically significant (*t* = 4.5610, *p* = 0.0061). Similarly, the period prevalence of paternal postpartum depression within the first month was 9.7% (95% CI 7.4–12.8%, *Q* = 14.84, *p* = 0.0216, *I*^*2*^ = 59.6%, tau^2^ = 0.0867) based on 7 included papers; a visual inspection of the funnel plot for this period revealed symmetry, and Egger’s regression test for funnel plot asymmetry was statistically nonsignificant (*t* = − 1.1231, *p* = 0.3124). The period prevalence of paternal postpartum depression during 1 to 3 months postpartum was 8.6% (95% CI 5.5–13.3%) based on one included paper. The period prevalence of paternal depression during 3 to 6 months postpartum was 13.2% (95% CI 11.6–15.0%, *Q* = 5.27, *p* =  0.1530, *I*^*2*^ = 43.1%, tau^2^ = 0.0098) based on 4 included papers; a visual inspection of the funnel plot revealed symmetry, and Egger’s regression test for funnel plot asymmetry was statistically nonsignificant (*t* = 1.5097, *p* = 0.2702). The period prevalence of paternal depression from 6 months to 1 year postpartum was 8.2% (95% CI 1.3–38.0%, *Q* = 54.16, *p* < 0.0001, *I*^*2*^ = 98.2%, tau^2^ = 1.8913) based on 2 included papers (Fig. 2).

**Fig. 3:**
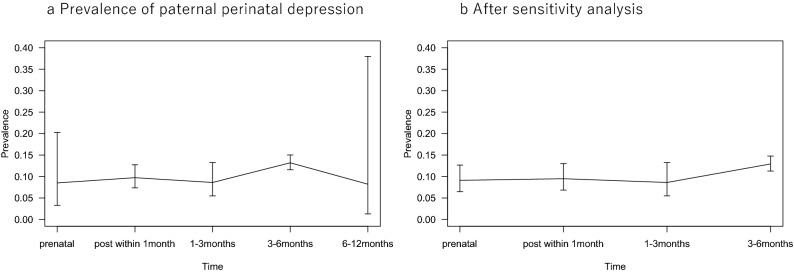
Time plots for the prevalence of paternal perinatal depression among Japanese men

High heterogeneity was observed in the prevalence data in the prenatal period and the 6–12 months postpartum period. The prevalence of perinatal depression among Japanese men peaked at 3–6 months after birth, and its overall prevalence was approximately 10%, as calculated using a random-effects model (Fig. 3a).

### Relative risk of perinatal depression by gender and test of heterogeneity

A subgroup analysis of gender differences in perinatal depression was performed.

**Fig. 4:**
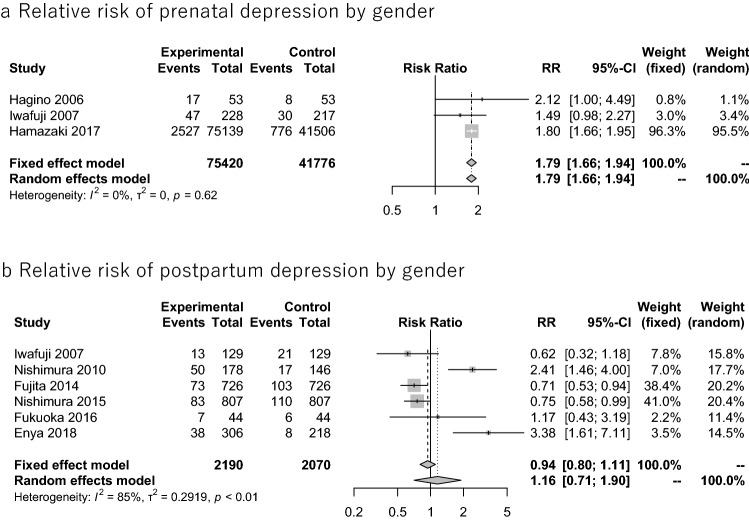
Forest plot for relative risk of perinatal depression by gender

We gathered studies that showed the prevalence of depression among men and women within the same paper. Then, we calculated the relative risks of perinatal depression among men and women presented in each paper, and we performed a meta-analysis on the relative risk. The results showed that women had a significantly higher prevalence of prenatal depression than men (relative risk = 1.79; 95% CI 1.66–1.94, *Q* = 0.95, *p* = 0.6223, *I*^*2*^ = 0.0%, tau^2^ = 0). However, there was no significant difference between men and women in the prevalence of postpartum depression (relative risk = 1.16; 95% CI 0.71–1.90, *Q* = 33.08, *p* < 0.0001, *I*^*2*^ = 84.9%, tau^2^ = 0.2919) (Fig. 4).

### Sensitivity analysis

A sensitivity analysis was performed to examine the robustness of the data by excluding individual studies and reanalyzing the remaining data. In particular, the analysis focused on heterogeneity. Studies included in our meta-analysis used the EPDS, CES-D, K6, and Self-Rating Depression Scale (SDS). We estimated that including these different measurements in the meta-analysis affected the heterogeneity of the results [4]. Previous reports have shown that the EPDS is not only the most widely used scale for evaluating perinatal depression in women, but also an accepted tool for assessing perinatal depression in men [4]. It is also known that the specificity of the EPDS is superior to that of other available scales in evaluating perinatal depression in men during the transition to parenthood [42]. For this reason, a sensitivity analysis was conducted by collecting only studies that used the EPDS.

The prevalence of prenatal or postpartum depression among men after the sensitivity analysis is presented below. The period prevalence of paternal prenatal depression was 9.1% (95% CI 6.5–12.7%, *I*^*2*^ = 0.0%), as determined from 2 included papers. Similarly, the period prevalence of paternal postpartum depression within the first month was 9.5% (95% CI 6.8–13.0%, *I*^*2*^ = 71.2%) based on 5 included papers, 8.6% (95% CI 5.5–13.3%) during 1–3 months postpartum based on one included paper, and 12.9% (95% CI 11.3–14.8%) during 3 to 6 months postpartum based on 3 included papers. There were no papers with data on the period from 6 months to 1 year postpartum after the sensitivity analysis. The prevalence of depression in every period exhibited low or moderate heterogeneity after the sensitivity analysis (Fig. 3b).

## Discussion

Our study revealed that the prevalence of perinatal depression among Japanese men was approximately 10%, with a peak at 3–6 months after birth. Another previous study reported that the prevalence of major depression among the general Japanese population was 2.9% [43]; thus, the prevalence of paternal perinatal depression was considerably higher than the prevalence of depression in the general population. This result is expected to have a significant impact on Japan's health policy and public health. Interestingly, similar to our findings, the latest international meta-analysis of prevalence of paternal perinatal depression also reported that the peak of prevalence occurred 3–6 months after childbirth [4]. However, there is no clear consensus as to why the peak of prevalence occurs in that period. Also, the difference in the prevalence of paternal perinatal depression between each time period was not statistically significant given that the 95% confidence intervals overlap for each time period.

The subgroup analysis revealed that females exhibited a significantly increased prevalence of depression during pregnancy than men did during their partners’ pregnancies. Interestingly, no statistically significant difference was noted between men and women regarding the prevalence of depression after childbirth. In other words, we found that men have postpartum depression at the same frequency as women do. Suicide in women due to postpartum depression is a social problem [8], but men may also succumb to suicide during the perinatal period because of depression [6]. Thus, the perinatal period is a time when the mental state of men requires attention. Men not only face stress due to role changes and social responsibilities in the family, but also may have reduced marital relationship satisfaction with women who have postpartum depression [38, 44]; thus, the prevalence of postpartum depression in couples may be similar.

We also found that the studies included in our meta-analysis exhibited high heterogeneity; therefore, sensitivity analysis was performed to examine the robustness of the data. The EPDS, the Center for Epidemiological Studies-Depression (CES-D) scale [45], SDS [46] and K6 [47] were used as evaluation scales for depression in our study. Notably, in our meta-analysis, K6 had two very different cutoff values. Based on previous research, the optimal cutoff value for the K6 for the evaluation of depression in Japanese community residents was 4/5 points, not 12/13, points because it led to fewer false-negative outcomes [28, 48]. Previous research reported that assessing depression with different rating scales is expected to result in variability [4]. Therefore, it was inferred that the mixture of the evaluation scales affected the heterogeneity. Among all measures to evaluate perinatal depression in women, the EPDS is the most frequently used worldwide [49]. After the extraction of studies that used the EPDS, the sensitivity analysis showed less than 75% heterogeneity among all time periods. The EPDS was developed by Cox [50], and Okano translated it into Japanese [51]. Regarding perinatal depression among Japanese women, some studies showed that the appropriate cutoff value for the EPDS was 8/9 in the postpartum period [51] and 11/12 during pregnancy [52]. On the other hand, regarding perinatal depression among Japanese men, it is reported that the appropriate cutoff value for the EPDS is 7/8 in the postpartum period [39]. However, there is insufficient evidence regarding the appropriate cutoff value for the EPDS in Japanese men during the prenatal period, and this issue requires further study.

A recent study reported that it was difficult to detect paternal postpartum depression [53]. Mental health problems in women could be detected in the maternal and child health care system. On the other hand, paternal perinatal depression is not well recognized in healthcare workers, and a screening and prevention system is lacking [13]. Thus, healthcare workers may have overlooked those who were seeking assistance. In addition, it has been suggested that a lack of knowledge of paternal postpartum depression may be a barrier to proper treatment [54]. How the attitudes of healthcare workers affect paternal perinatal depression is an important issue. In Japan, several studies have actively interviewed men about their mental state, but it has not been established as a general screening system. We recommend screening for paternal perinatal depression. However, unfortunately, it is currently rare for men to participate with women in the maternal and child health care system. Thus, it is difficult for healthcare professionals to create a system for direct screening of paternal perinatal depression. Therefore, indirect assessments of paternal perinatal depression are being studied. A previous study in Japan suggested that the effectiveness of the K6, K10 and PHQ-9 may be compromised when used by female partners to assess the perinatal depression of their male partners [26]. In addition, another previous study in the United States developed the Edinburgh Postnatal Depression Scale-Partner (EPDS-P) for perinatal women to assess depression in their male partners [55]. This study revealed that the EPDS-P demonstrated good sensitivity and negative predictive value in indirect identifying paternal perinatal depression [55]. It may be worth investigating the accuracy of indirect screening for paternal perinatal depression in Japanese men using EPDS-P based on studies in the United States. After indirect screening for paternal perinatal depression, providing personalized support to high-risk men was considered superior to direct screening in terms of feasibility. In Japan, the participation rate of women for infant health checkups at 1 month postpartum is high at 86.4% [56]. Therefore, we suggest developing a system to evaluate paternal and maternal mental health simultaneously at this point based on women’s assessment.

Given the similarities between the results of our study and a prior international meta-analysis [4], paternal perinatal depression may occur regardless of cultural or regional differences. With the birth of children, family relationships change; men become more financially and socially responsible for their family and are expected to help their partner and children [30, 57–59]. These social changes occur regardless of cultural differences and place psychological pressure on men. Thus, factors that can occur regardless of cultural differences may be more involved in perinatal depression among men than factors that differ by country or culture. These hypotheses are not supported by concrete findings and require further consideration as indicators for future research.

On the other hand, international comparisons may provide useful insights into the management of perinatal depression in men. Importantly, an international comparison meta-analysis found no significant difference in the prevalence of paternal perinatal depression in South America, Asia, Australia or Oceania compared to North America [4]. However, the prevalence of paternal perinatal depression was found to be significantly lower in Europe, including the United Kingdom, than in North America [4]. The percentage of men who receive parental leave is higher in Europe than in other regions, which may help reduce stress in European men during the perinatal period [4]. Japan may also reduce the prevalence of paternal perinatal depression by creating a system that greatly facilitates parental leave for men. In Japan, the rate of parental leave among mothers 83.2%, while the rate among fathers is only 5.14% [60]. A review of the work-life balance of the child-rearing generation may lead to the prevention of paternal perinatal depression. These points are particularly relevant to a Japanese readership.

## Limitations

This study has several limitations. The assessment of depression in our study relied on self-administered screening tools and was not based on structured interviews or physician diagnoses. This limitation leaves the possibility of the presence of mood disorders other than depression, such as bipolar disorder. However, the importance of this study is not impaired, as screening by midwives and public health nurses will also be a gateway to appropriate care for depressed patients. Furthermore, in this meta-analysis, heterogeneity was high because of the use of various evaluation scales and different cutoff values. In addition, the number of surveys conducted within each period was insufficient, particularly after the sensitivity analysis. In order to address these limitations, it is necessary to conduct further research after obtaining consensus on the evaluation scale and the cutoff value. Obtaining more robust basic data will contribute to Japanese public health. It was difficult to extract raw data about whether participants received treatment and whether they had a family history of mood disorder. However, a recent study reported that paternal postpartum depression may correlate with offspring depression at 18 years of age [61]. Therefore, a potentially important topic for future research will be whether perinatal depression in Japanese men is associated with a history of perinatal depression in their parents.

## Conclusions

The prevalence of perinatal depression among Japanese men peaked 3–6 months after birth, and its overall prevalence was approximately 10%. These results are similar to those of an international meta-analysis of perinatal depression. In Japan, the prevalence of perinatal depression among men was significantly higher than the prevalence of major depression in the general population (2.9%). Notably, we revealed that the prevalence of postpartum depression among the Japanese population was as high in men as in women. Therefore, it is suggested that healthcare workers be more watchful for paternal perinatal depression in the postpartum period than in the prenatal period.

## Data Availability

All data generated or analyzed during this study are included in this published article.
